# Effects of feed intake-regulated nutritional compensation strategies on growth performance, blood indices, and rumen microbiota of Gangba sheep

**DOI:** 10.1016/j.aninu.2026.04.005

**Published:** 2026-08-04

**Authors:** Yining Xie, Xiaokang Jing, Zhaohan Zhan, Liang Chen, Hongfu Zhang

**Affiliations:** a State Key Laboratory of Animal Nutrition and Feeding, Key Laboratory of Animal Nutrition and Feed Science of Ministry of Agriculture and Rural Affairs, Institute of Animal Science, Chinese Academy of Agricultural Sciences, Beijing 100193, China; b Animal Sciences, Gembloux Agro-Bio Tech, TERRA Teaching and Research Centre, University of Liège, Gembloux 5030, Namur, Belgium

**Keywords:** Compensatory growth, Feed restriction, Apparent digestibility, Feed-to-gain ratio, Rumen microbiota, Gangba sheep

## Abstract

Feed intake-regulated nutritional compensation improves feed efficiency in sheep; however, its relevance and underlying mechanisms in high-altitude indigenous breeds such as Gangba sheep remain unclear. Ninety-six four-month-old healthy male Gangba sheep (16.20 ± 0.61 kg) were randomly assigned to four groups (six replicates per group, four sheep per replicate): ad libitum feeding (ALF) and low-, medium-, and high-restriction feeding groups (LRF, MRF, and HRF), receiving 85%, 70%, and 55% of ALF intake, respectively, for 30 d, followed by 60 d of compensatory refeeding. During the restriction period, increasing restriction intensity decreased body weight at d 30, average daily feed intake, and average daily gain (*P* < 0.05) and increased the feed-to-gain ratio (*P* < 0.001). Medium and high restriction also decreased total antioxidant capacity (*P* = 0.010), immunoglobulin A (*P* < 0.001), immunoglobulin M (*P* = 0.045), neutral detergent fiber digestibility (*P* = 0.004), ruminal papilla height (*P* = 0.006), and ruminal butyric acid concentration (*P* = 0.013). Over the whole experimental period, feed intake-regulated nutritional compensation significantly reduced average daily feed intake compared with ALF (*P* < 0.001). The feed-to-gain ratio was lower in the LRF group than in the ALF group (*P* = 0.004), indicating improved feed efficiency under low restriction feeding. Restriction feeding also induced adaptive changes in gastrointestinal development and rumen microbial diversity, with microbial α diversity recovering after refeeding while community structure remained differentiated among treatments. Overall, low feed restriction (85% of ALF) enhanced feed utilization while maintaining physiological homeostasis in Gangba sheep under forage-limited high-altitude conditions.

## 1 Introduction

Gangba sheep are a locally adapted subtype of Tibetan sheep inhabiting the Qinghai–Tibetan Plateau. This region experiences chronic forage shortages, prolonged cold seasons, and harsh environmental conditions, which severely limit livestock productivity. Living under persistent feed scarcity and extreme hypoxic and cold stress, Gangba sheep have developed long-term physiological and microbiological adaptations to low temperatures, low oxygen concentration, and low-quality forages (Zhang et al., 2021, 2024). These characteristics confer strong regional specificity and make Gangba sheep a representative high-altitude ruminant model for evaluating feeding strategies aimed at improving production efficiency in forage-limited plateau systems.

Compensatory growth, defined as accelerated growth after a period of nutritional restriction, is a well-recognized physiological mechanism in ruminants (Hornick et al., 2000; Mahyuddin, 2004). It is closely associated with improved feed efficiency, altered nutrient partitioning, and metabolic regulation (Hornick et al., 2000; Hu et al., 2019). Among various nutritional manipulation strategies, feed-intake regulation is of particular interest because of its operational simplicity and its practical relevance in regions with pronounced seasonal variation in forage availability. Previous studies have shown that sheep can achieve partial or full compensatory growth after nutritional restriction (Ding et al., 2016; Kamalzadeh and Aouladrabiei, 2009; Neto et al., 2011). Therefore, identifying how different levels of feed restriction influence compensatory responses is essential for developing sustainable and cost-effective feeding programs in high-altitude areas of the Qinghai-Tibetan Plateau. In the present study, “feed intake-regulated nutritional compensation” is defined as a feeding strategy involving a controlled period of quantitative feed restriction followed by a compensatory refeeding phase, with the objective of improving feed utilization efficiency while maintaining physiological homeostasis. Unlike traditional compensatory growth approaches that mainly emphasize rapid body weight recovery after undernutrition, this strategy highlights the regulation of feed intake level as a practical nutritional management tool to optimize growth performance, metabolic adaptation, and resource use efficiency under forage-limited high-altitude conditions.

Feed restriction induces a series of physiological responses in sheep, including alterations in energy metabolism, oxidative balance, immune function, and hormonal regulation (Keogh et al., 2015; Zych et al., 2019). Serum biochemical and antioxidant parameters serve as sensitive indicators of metabolic stress and redox status, whereas immune-related markers reflect the host's ability to cope with nutritional challenges (Wang et al., 2022; Zeng et al., 2021). Nutrient digestibility and gastrointestinal morphology—such as ruminal papillae and intestinal villi—are highly responsive to changes in energy supply and fermentation-derived metabolites (e.g., butyrate), which directly affect the absorption of volatile fatty acids (VFAs) and other nutrients (Li et al., 2023; Penner et al., 2011).

The rumen microbiota plays a central role in mediating host adaptation to feed restriction and subsequent refeeding by regulating fermentation, fiber degradation, nitrogen recycling, and energy production (Malmuthuge and Guan, 2017; Xue et al., 2020). While microbial responses to undernutrition or controlled feeding have been reported in various ruminant species, little is known about stage-specific microbial adaptations during the end of restriction and the end of refeeding in high-altitude indigenous breeds. Considering that Gangba sheep have evolved a unique digestive physiology and microbial ecosystem under long-term ecological pressure, characterizing their physiological, digestive, and microbial responses to restriction and refeeding is of substantial scientific and practical relevance.

To date, no study has comprehensively evaluated growth performance, serum physiological characteristics, nutrient digestibility, gastrointestinal morphology, rumen fermentation patterns, and full-length 16S rRNA-based rumen microbial profiles across the entire restriction-refeeding cycle in Gangba sheep. This knowledge gap limits the development of evidence-based feeding strategies tailored for high-altitude farming systems. Based on previous findings in conventional sheep breeds, it was hypothesized that varying levels of feed restriction would induce distinct effects on growth, physiological homeostasis, digestive function, and rumen microbial structure. Therefore, compensatory responses of Gangba sheep during refeeding were expected to be modulated by the severity of prior restriction, with each restriction level triggering unique physiological, digestive, and microbial adjustments during both restriction and refeeding. This study aims to elucidate these mechanisms and provide a scientific basis for designing sustainable and efficient nutritional strategies for local sheep in forage-deficient high-altitude regions.

## 2 Material and methods

### 2.1 Animal ethics statement

All experimental procedures were approved by the Laboratory Animal Welfare and Ethics Committee of the Institute of Animal Science, Chinese Academy of Agricultural Sciences (approval No. IAS2024–126; approval date: January 26, 2024) and were performed in accordance with the guidelines for the care and use of experimental animals issued by the Ministry of Agriculture and Rural Affairs of China (MARA, 2019).

### 2.2 Experimental design and husbandry management

A total of 96 four-month-old male Gangba sheep (initial body weight: 16.20 ± 0.61 kg) were used. Sheep were blocked by body weight and randomly assigned to four dietary treatments, each consisting of six replicates (pens) with four sheep per replicate: ad libitum feeding (ALF), low restricted feeding (85% of ALF, LRF), medium restricted feeding (70% of ALF, MRF), and high restricted feeding (55% of ALF, HRF). The experiment lasted 104 d, including a 14 d adaptation period, a 30 d restricted feeding period, and a 60 d compensatory feeding period.

Feed was offered twice daily at 09:00 and 17:00, and daily refusals were recorded to calculate average daily feed intake (ADFI). Feed intake was recorded on a pen basis (four sheep per pen during the restriction phase and three sheep per pen during the compensatory feeding phase), and ADFI was calculated using pen as the experimental unit. During the restriction phase, sheep in the ALF group were fed ad libitum, whereas the restricted groups received predetermined proportions of the ALF intake based on the recorded intake of the previous day. During the compensatory feeding phase, all sheep were returned to ad libitum feeding. All animals were fed a complete pelleted diet formulated according to the nutrient requirements for growing sheep (NRC, 2007). The ingredients and nutrient levels of the diet are shown in Table 1. Clean drinking water was provided ad libitum throughout the entire experiment.

**Table 1:** Ingredients and nutrient levels of the diet (%, as DM basis).

Ingredients	Content	Nutrients^2^	Content
Oat hay	52.0	Crude protein	12.77
Corn	28.0	Ether extract	1.57
Soybean meal	13.0	Neutral detergent fiber	38.75
Wheat bran	5.0	Acid detergent fiber	22.37
Limestone	0.5	Organic matter	92.91
Premix^1^	1.0	Metabolizable energy, MJ/kg	10.14
Salt	0.5		
Total	100.0		

DM = dry matter.

1 Per kg of premix contained: vitamin A 700,000 to 1,000,000 IU, vitamin E 4000 IU, vitamin K_3_ 100 mg, vitamin B_1_ 100 mg, vitamin B_2_ 260 mg, vitamin B_12_ 1000 μg, vitamin B_6_ 100 mg, vitamin D_3_ 200,000 to 500,000 IU, niacin 1600 mg, pantothenic acid 500 mg, folic acid 50 mg, copper 0.2 to 1.5 g (as CuSO_4_·5H_2_O), iron 3 to 20 g (as FeSO_4_·7H_2_O), zinc 2 to 12 g (as ZnSO_4_·H_2_O), manganese 2 to 14 g (as MnSO_4_·H_2_O), iodine 10 to 100 mg (as KI), selenium 10 to 50 mg (as Na_2_SeO_3_), cobalt 5 to 40 mg (as CoSO_4_·7H_2_O).

2 Organic matter was calculated as DM minus ash. Metabolizable energy was calculated from ingredient composition according to NRC (2007). The others were measured values.

### 2.3 Growth performance and apparent digestibility of nutrients

Body weight was recorded individually on d 0, 30 (end of feed restriction), and 90 (end of compensatory refeeding). Average daily gain (ADG) and feed-to-gain ratio (F:G) were calculated for the restriction and refeeding periods. During the final 7 consecutive days of each period, one sheep per pen was randomly selected and fitted with a harness-mounted fecal collection bag to facilitate grab (representative) fecal sampling rather than total fecal collection (*n* = 6) (Sun et al., 2016; Tekliye et al., 2018). Fecal samples were collected during two fixed sampling windows each day (4 h after the morning feeding and 4 h after the afternoon feeding) and combined to obtain a daily composite sample for apparent nutrient digestibility estimation using acid-insoluble ash (AIA) as an internal marker (Sales and Janssens, 2003; Zeng et al., 2023). Approximately 20% of the collected feces was subsampled, immediately acidified with 0.5% H_2_SO_4_, and stored at −20 °C. Samples were pooled at the end of collection for analysis (Ren et al., 2018).

### 2.4 Slaughter procedures and sample collection

At the end of each phase, six sheep per treatment (one lamb per replicate) were randomly selected and fasted for 24 h prior to blood and tissue collection. Blood samples were collected via the jugular vein into 10-mL vacuum tubes, centrifuged at 3000 × *g* for 15 min, and serum was stored at −80 °C. Sheep were then slaughtered in a commercial abattoir following MARA (2019) regulations. Rumen contents were collected immediately after evisceration. Rumen pH was measured in situ using a portable pH meter (HI8424, Hanna Instruments, Woonsocket, RI, USA). Approximately 8 mL of rumen content was collected for microbial DNA extraction. The remaining portion was filtered through four layers of fine gauze and centrifuged at 3000 × *g* for 30 min at 4 °C to obtain rumen fluid. Two 50 mL aliquots of rumen fluid were collected for analysis of VFAs. Additionally, rumen epithelium (1.5 cm × 1.5 cm) and jejunal tissue (1 cm) were sampled and fixed in 4% paraformaldehyde for histological examination. All samples were snap-frozen on dry ice and stored at −80 °C until analysis (Li et al., 2023).

### 2.5 Serum analysis

Serum indicators were analyzed according to standard procedures (Xie et al., 2025). Biochemical parameters, including alanine aminotransferase (ALT), aspartate aminotransferase (AST), triglycerides (TG), total cholesterol (TC), high-density lipoprotein (HDL), and low-density lipoprotein (LDL), were measured using an AU680 automatic biochemical analyzer (Beckman Coulter, Inc., Brea, CA, USA). Antioxidant indices, including total antioxidant capacity (T-AOC; Cat. No. JN24358X), glutathione peroxidase (GSH-Px; Cat. No. JN24284X), malondialdehyde (MDA; Cat. No. JN24318X), and superoxide dismutase (SOD; Cat. No. JN24918X), were determined using commercial assay kits (Shanghai Jining Biotechnology Co., Ltd., Shanghai, China) according to the manufacturer's instructions. Serum immunoglobulins and cytokines were quantified using sheep-specific ELISA kits (Shanghai Jining Biotechnology Co., Ltd., Shanghai, China), including immunoglobulin A (IgA; Cat. No. JN82299), immunoglobulin G (IgG; Cat. No. JN82597), immunoglobulin M (IgM; Cat. No. JN20997), tumor necrosis factor-α (TNF-α; Cat. No. JN20906), interleukin-2 (IL-2; Cat. No. JN22035), interleukin-4 (IL-4; Cat. No. JN21766), interleukin-6 (IL-6; Cat. No. JN21752), and interferon-γ (IFN-γ; Cat. No. JN20780). Growth hormone axis-related hormones were also determined using sheep-specific ELISA kits (Shanghai Jining Biotechnology Co., Ltd., Shanghai, China), including growth hormone (GH; Cat. No. JN83713), growth hormone-releasing hormone (GHRH; Cat. No. JN81703), and insulin-like growth factor-1 (IGF-1; Cat. No. JN81107).

### 2.6 Chemical analysis

Feed and fecal samples were dried at 65 °C for 48 h, ground, and sieved through a 40-mesh screen. Dry matter (DM; method 930.15) and ash (method 975.03) were determined according to AOAC (2012). Crude protein (CP; method 984.13) was determined using a Kjeltec 8400 automatic Kjeldahl analyzer (FOSS Analytical A/S, Hillerød, Denmark), and ether extract (EE; method 920.39) was determined using a Soxtec automated fat extraction system (FOSS Analytical A/S, Hillerød, Denmark). Neutral detergent fiber (NDF) and acid detergent fiber (ADF) were analyzed using α-amylase-assisted detergent procedures (Soest et al., 1991). Metabolizable energy (ME) was calculated from the ingredient composition and tabulated ME values according to NRC (2007). The content of OM was calculated using the formula:

OM(%)=100-Ash(%).

Acid-insoluble ash (AIA) content in diet and fecal samples was determined as described (Sales and Janssens, 2003; Zeng et al., 2023).

### 2.7 Histological analyses

#### 2.7.1 Rumen morphology

Rumen epithelium samples were fixed in 10% neutral-buffered formalin, dehydrated through graded ethanol, cleared in xylene, and embedded in paraffin. Sections (6 μm) were stained with hematoxylin and eosin (H&E). Papilla length, width, and surface area were measured using a stereomicroscope (SteREO Discovery.V12, Carl Zeiss Microscopy GmbH, Jena, Germany) and AxioVision software (version 4.6, Carl Zeiss Microscopy GmbH, Jena, Germany) following established protocols (Lesmeister et al., 2004; Li et al., 2023).

#### 2.7.2 Jejunal morphology

Jejunal samples were embedded in paraffin, sectioned at 3 to 4 μm using a rotary microtome (RM 2235, Leica biosystems Nussloch GmbH, Nussloch, Germany), and stained with H&E. Four non-overlapping fields per sample were examined under a light microscope (Axioskop, Carl Zeiss Microscopy GmbH, Jena, Germany). Villus height (VH), crypt depth (CD), and VH/CD were calculated according to standard procedures (Lesmeister et al., 2004; Li et al., 2023).

### 2.8 Rumen fermentation parameters and enzyme activities

Rumen fluid samples were centrifuged, and the clarified supernatants were collected for rumen fermentation parameter and enzyme activity determinations. Rumen ammonia nitrogen concentration was determined by UV-visible spectrophotometry at 620 nm using a UV-2450 spectrophotometer (Shimadzu Corporation, Kyoto, Japan). Individual VFAs, including acetic, propionic, iso-butyric, butyric, valeric, and iso-valeric acids, were quantified using a 7890A gas chromatograph equipped with an HP-FFAP column (Agilent Technologies, Santa Clara, CA, USA). Rumen fluid samples were acidified with phosphoric acid and centrifuged, and the resulting supernatants were extracted with butanol containing 2-ethylbutyric acid as an internal standard. Helium was used as the carrier gas. The oven temperature was programmed from 80 to 120 °C at 20 °C/min, then to 160 °C at 5 °C/min, and finally to 220 °C, where it was held for 3 min. Quality-control samples were analyzed after every six samples, and the relative standard deviations of target compounds were maintained below 15%.

All enzyme activities were determined using commercial assay kits supplied by Shanghai Jining Biotechnology Co., Ltd. (Shanghai, China), according to the manufacturer's instructions. Absorbance was measured using a microplate reader (PowerWave X, BioTek Instruments, Inc., Winooski, VT, USA). Protease activity was expressed as neutral protease activity and determined using a neutral protease assay kit (JN24383X), based on the amount of tyrosine released from casein hydrolysis under neutral conditions, with absorbance read at 680 nm. Lipase activity was determined using a lipase assay kit (JN24761X) based on the p-nitrophenol method, which quantifies p-nitrophenol released from the hydrolysis of p-nitrophenyl palmitate, with absorbance read at 405 nm. Cellulase activity was determined using a cellulase assay kit (JN24574X) and the 3,5-dinitrosalicylic acid (DNS) method, based on the reducing sugars released from sodium carboxymethyl cellulose degradation, with absorbance read at 540 nm. α-Amylase activity was determined using an α-amylase assay kit (JN24263X) and the DNS method, based on the reducing sugars released during starch hydrolysis, with absorbance read at 540 nm. Lysozyme was determined using a lysozyme assay kit (JN62598X) and a turbidimetric method by monitoring the decrease in turbidity of the bacterial suspension, with absorbance read at 530 nm.

### 2.9 Full-length 16SrRNA gene sequencing

Total microbial DNA was extracted from rumen content samples using the E.Z.N.A. Soil DNA Kit (Omega Bio-Tek, Norcross, GA, USA) following the manufacturer's instructions. DNA quality and integrity were assessed by 1% agarose gel electrophoresis and quantified using a NanoDrop 2000 spectrophotometer (Thermo Fisher Scientific Inc., Waltham, MA, USA). The bacterial 16S rRNA gene was amplified with barcoded primers 27F (5′-AGRGTTYGATYMTGGCTCAG-3′) and 1492R (5′-RGYTACCTTGTTACGACTT-3′), and libraries were constructed using the SMRTbell Prep Kit 3.0 (Pacific Biosciences, Menlo Park, CA, USA) (Johnson et al., 2019; Schloss et al., 2016). Raw reads were demultiplexed and quality-filtered prior to downstream analysis. High-quality amplicon sequence variants (ASVs) were generated using the DADA2 algorithm implemented in the QIIME2 platform (v2021.11) (Callahan et al., 2016). To minimize sequencing depth bias, all samples were rarefied to 6000 sequences per sample, with Good's coverage reaching 99.09%. Taxonomic assignment of ASVs was performed against the SILVA 16S rRNA reference database (release 138) using the QIIME2 feature-classifier plugin based on the BLAST algorithm (Quast et al., 2013). Alpha diversity indices (Ace, Chao, Shannon) were calculated using Mothur (Schloss et al., 2009). Beta diversity was assessed using Bray–Curtis distances, and differences in microbial community structure among treatments were evaluated by PERMANOVA (Anderson, 2008). Differential taxa were identified using linear discriminant analysis effect size (LEfSe) (Segata et al., 2011). Relative abundances of microbial taxa were tested for normality using the Shapiro-Wilk test. As most compositional data were not normally distributed, treatment differences were evaluated using nonparametric Kruskal-Wallis tests, and *P*-values were adjusted using a false discovery rate (FDR) correction based on the Benjamini-Hochberg procedure. In addition, Spearman's rank correlation analysis was performed to evaluate the associations between rumen microbial taxa (species level) and rumen fermentation parameters and enzyme activities.

### 2.10 Calculations and statistical analysis

The calculation formula for apparent digestibility using AIA is as follows:

Apparentdigestibility(%)=100×[1-(Nutrientcontentinfeces×AIAcontentindiet)/(Nutrientcontentindiet×AIAcontentinfeces)].

The experimental data were examined utilizing SAS 9.4 (SAS Institute Inc., Cary, NC, USA) with the PROC MIXED model. The experiment followed a randomized complete block design, in which sheep were blocked by initial body weight before random allocation to treatments, and block was treated as a random effect in the mixed model. Although feed restriction treatments were applied at the pen level, body weight was measured individually and analyzed using individual animals as observational units, with pen (replicate) nested within treatment and block (initial body weight grouping) included as random effects to account for potential clustering. Intake-related variables (e.g., ADFI and F: G) were calculated on a pen basis and analyzed using pen as the experimental unit. Because growth performance variables were recorded at multiple experimental phases (restriction and compensatory feeding), experimental phase was included as a repeated fixed effect, and repeated measurements of body weight were modeled as observations within animal over time. Treatment, phase, and their interaction were specified as fixed effects, with animal defined as the subject term. Appropriate covariance structures (e.g., compound symmetry, first-order autoregressive, and unstructured) were evaluated. The structure with the lowest Akaike information criterion (AIC) was selected. Degrees of freedom were adjusted using the Kenward–Roger method (Kenward and Roger, 1997). Body weight measured at different experimental phases (e.g., d 0, 30, and 90) was therefore treated as a repeated measure. Intake-related variables were summarized at the pen level within each phase and were not analyzed as repeated measures. For serum indices, nutrient digestibility, gastrointestinal morphology, rumen fermentation parameters, enzyme activities, and microbial diversity analyses, different sheep were randomly selected at each sampling phase; therefore, these variables were analyzed as independent observations rather than repeated measures. The beneficial effects of nutritional compensation strategy on Gangba sheep were evaluated by comparing the ALF with three restricted feed treatment groups, as well as by assessing linear and quadratic effects. Additionally, least-squares means were compared using the Tukey–Kramer adjustment for multiple comparisons. Differences were considered significant at *P* ≤ 0.05 and trends were declared at 0.05 ＜ *P* < 0.10.

The PROC MIXED model included both random and fixed effects and was expressed as follows:

$$Y_{ijkl}=\mu +B_{i}+T_{j}+Pk_{\left(j\right)}+T_{l}+{\left(T\times T_{l}\right)}_{jl}+\varepsilon_{ijkl}\text{,}$$

where *Y*_*ijkl*_ is the dependent variable; *μ* is the overall mean; *B*_*i*_ is the random effect of block (initial body weight grouping); *T*_*j*_ is the fixed effect of nutritional compensation strategy (*j* = 55%, 70%, 85%, and ad libitum feeding); *Pk*_*(j)*_ is the random effect of pen nested within treatment; *T*_*l*_ is the fixed repeated effect of experimental phase (restriction vs compensatory feeding); (*T* × *T*_*l*_)_*jl*_ is the interaction between treatment and phase, and *ε*_*ijkl*_ is the residual error term.

## 3 Results

### 3.1 Growth performance

During the restricted feeding period (d 1-30), increasing restriction levels resulted in a linear decrease in body weight at d 30, ADFI, and ADG (*P* < 0.05; Table 2). The F: G increased with restriction intensity and displayed a quadratic effect (*P* = 0.033).

**Table 2:** Effects of feed intake-regulated nutritional compensation strategies on the growth performance in Gangba sheep.

Item	Groups^1^					*P*-value		
	ALF	LRF	MRF	HRF	SEM	Treatment	Linear	Quadratic
**Restricted feeding period**								
BW at d 1, kg	16.33	16.15	16.19	16.31	0.113	0.963	0.961	0.706
BW at d 30, kg	20.91^a^	20.19^b^	19.06^c^	18.06^d^	0.264	<0.001	<0.001	0.820
ADFI, g/d	1180.56^a^	1018.06^b^	837.50^c^	672.19^d^	12.813	<0.001	<0.001	0.723
ADG, g/d	152.78^a^	133.33^b^	95.83^c^	59.48^d^	6.584	<0.001	<0.001	0.214
F:G	7.77^b^	7.90^b^	8.89^b^	11.41^a^	0.524	<0.001	<0.001	0.033
**Compensatory feeding period**								
BW at d 90, kg	30.70	30.67	28.63	27.34	1.032	0.209	0.956	0.421
ADFI, g/d	1298.55	1262.85	1214.81	1303.01	34.094	0.262	0.903	0.530
ADG, g/d	163.34	176.85	172.75	158.40	16.341	0.183	0.512	0.040
F:G	8.02^a^	6.89^b^	7.56^ab^	8.01^a^	0.271	0.022	0.586	0.008
**Whole feed period (d 1–90)**								
ADFI, g/d	1251.14^a^	1168.89^b^	1092.24^c^	1004.60^d^	17.842	<0.001	<0.001	0.882
ADG, g/d	159.58^a^	157.78^a^	137.99^b^	119.67^c^	14.183	<0.001	<0.001	0.013
F:G	7.84^b^	7.41^c^	7.91^b^	8.44^a^	0.401	0.004	0.006	<0.001

BW = body weight; ADFI = average daily feed intake; ADG = average daily gain; F:G = feed-to-gain ratio; SEM = standard error of the mean.

Values within a row with no common superscripts differ significantly (*P* < 0.05). BW and ADG were based on individual lambs (*n* = 24 during the restricted-feeding period and *n* = 18 during the compensatory-feeding and whole-feeding periods), whereas ADFI and F:G were based on six pens per treatment (*n* = 6).

1 ALF, ad libitum feeding; LRF, low restricted feeding; MRF, medium restricted feeding; HRF, high restricted feeding.

During compensatory feeding (d 31–90), F: G differed among treatments (*P* = 0.022). The LRF group exhibited a lower F:G compared with ALF (*P* = 0.022), and F:G demonstrated a quadratic effect with increasing restriction (*P* = 0.008). Although ADG did not differ among treatments, a quadratic effect was observed (*P* = 0.040).

Across the entire period (d 1–90), ADFI decreased linearly with restriction level (*P* < 0.001). ADG was lower in the MRF and HRF groups than in the ALF and LRF groups (*P* < 0.001) and exhibited a quadratic response to restriction level (*P* = 0.013). The overall F: G differed among treatments and showed both linear and quadratic effects (*P* < 0.05). Specifically, the F: G decreased by 5.48% in LRF group, while it increased by 0.89% and 7.65% in the MRF and HRF groups (*P* = 0.004), respectively.

### 3.2 Serum biochemical and antioxidant indices

During restriction, serum T-AOC decreased in MRF and HRF compared with ALF and LRF (*P* = 0.010; Table 3). MDA concentration was higher in the HRF group than in the ALF and MRF groups (*P* = 0.050), with a linear effect of restriction level (*P* = 0.022). SOD activity was lower in the MRF and HRF groups than in the LRF group (*P* = 0.024), accompanied by a linear decline with increasing restriction level (*P* = 0.004). During compensatory feeding, none of the biochemical or antioxidant indices differed significantly among treatments (*P* > 0.05).

**Table 3:** Effects of feed intake-regulated nutritional compensation strategies on serum biochemical and antioxidant indicators in Gangba sheep.

Item	Groups^1^					*P*-value		
	ALF	LRF	MRF	HRF	SEM	Treatment	Linear	Quadratic
**Restricted feeding period**								
Alanine aminotransferase, U/L	24.23	26.22	25.50	20.99	5.832	0.932	0.693	0.584
Aspartate aminotransferase, U/L	100.99	104.63	108.86	117.05	7.253	0.455	0.122	0.757
Triglycerides, mmol/L	1.59	1.57	1.68	1.56	0.121	0.875	0.952	0.664
Total cholesterol, mmol/L	0.37	0.38	0.31	0.31	0.041	0.419	0.148	0.915
High density lipoprotein, mmol/L	0.55	0.62	0.54	0.77	0.071	0.088	0.064	0.269
Low density lipoprotein, mmol/L	0.40	0.50	0.51	0.47	0.042	0.313	0.278	0.126
Total antioxidant capacity, μmol Trolox/mL	1.59^a^	1.60^a^	1.40^b^	1.37^b^	0.054	0.010	0.003	0.667
Glutathione peroxidase, U/mL	21.64	16.88	28.78	24.08	6.642	0.649	0.524	0.997
Malondialdehyde, nmol/mL	0.61^b^	0.96^ab^	0.76^b^	1.37^a^	0.181	0.050	0.022	0.532
Superoxide dismutase, U/mL	16.98^ab^	20.92^a^	12.29^b^	12.47^b^	2.084	0.024	0.004	0.377
**Compensatory feeding period**								
Alanine aminotransferase, U/L	38.13	35.87	30.27	34.27	7.231	0.888	0.601	0.670
Aspartate aminotransferase, U/L	149.17	152.80	154.90	147.00	10.272	0.948	0.925	0.581
Triglycerides, mmol/L	0.33	0.34	0.30	0.27	0.033	0.437	0.144	0.567
Total cholesterol, mmol/L	1.54	1.49	1.56	1.44	0.124	0.904	0.690	0.748
High density lipoprotein, mmol/L	0.63	0.60	0.62	0.63	0.084	0.437	0.144	0.567
Low density lipoprotein, mmol/L	0.52	0.51	0.57	0.54	0.051	0.825	0.690	0.748
Total antioxidant capacity, μmol Trolox/mL	2.50	2.57	2.40	2.48	0.061	0.211	0.351	0.999
Glutathione peroxidase, U/mL	199.81	192.85	172.81	184.21	5.862	0.113	0.265	0.563
Malondialdehyde, nmol/mL	1.07	0.77	0.79	0.76	0.143	0.374	0.160	0.379
Superoxide dismutase, U/mL	46.83	47.60	43.28	44.43	5.084	0.890	0.545	0.808

SEM = standard error of the mean.

Values within a row with no common superscripts differ significantly (*P* < 0.05). All values are based on six lambs per treatment (*n* = 6).

1 ALF, ad libitum feeding; LRF, low restricted feeding; MRF, medium restricted feeding; HRF, high restricted feeding.

### 3.3 Serum immune and hormonal indices

During the restricted-feeding period, serum IgA levels were lower in the MRF and HRF groups than in the ALF and LRF groups (*P* < 0.001), with a significant linear decrease as restriction level increased (*P* < 0.001; Table 4). Similarly, serum IgM levels were lower in the MRF and HRF groups than in the ALF and LRF groups (*P* = 0.045) and decreased linearly with increasing restriction level (*P* = 0.007). Conversely, serum GHRH concentration was higher in the HRF group than in the LRF group (*P* = 0.046) and increased linearly with increasing restriction level (*P* = 0.009). Besides, during compensatory feeding, no immune or hormonal parameters differed among groups (*P* > 0.05).

**Table 4:** Effects of feed intake-regulated nutritional compensation strategies on serum immune and hormonal indicators in Gangba sheep.

Item	Groups^1^					*P*-value		
	ALF	LRF	MRF	HRF	SEM	Treatment	Linear	Quadratic
**Restricted feeding period**								
Immunoglobulin A, g/L	16.70^a^	16.54^a^	13.25^b^	11.63^b^	0.841	<0.001	<0.001	0.383
Immunoglobulin G, g/L	13.62	13.27	14.21	13.09	0.742	0.727	0.849	0.612
Immunoglobulin M, g/L	1.31^a^	1.25^a^	1.17^b^	1.01^b^	0.071	0.045	0.007	0.510
Tumor necrosis factor-α, ng/L	372.78	458.97	274.64	284.65	62.153	0.158	0.122	0.547
Interleukin 2, pg/mL	132.14	157.98	204.28	115.66	28.491	0.172	0.981	0.058
Interleukin 4, pg/mL	14.15	15.77	18.35	18.03	2.374	0.565	0.196	0.687
Interleukin 6, pg/mL	622.17	636.64	510.62	415.58	84.402	0.242	0.062	0.524
Interferon -γ, pg/L	125.10	116.11	114.88	89.54	26.111	0.795	0.367	0.758
Growth hormone, ng/mL	5.98	7.25	8.25	8.05	1.152	0.508	0.177	0.528
Growth hormone-releasing hormone, pg/mL	11.24^b^	10.67^b^	15.58^ab^	19.17^a^	2.232	0.046	0.009	0.362
Insulin-like growth factor-1, ng/mL	155.45	127.17	127.87	144.84	24.053	0.805	0.775	0.358
**Compensatory feeding period**								
Immunoglobulin A, g/L	14.24	14.72	13.46	13.22	2.241	0.961	0.670	0.874
Immunoglobulin G, g/L	14.30	14.37	13.42	16.26	2.653	0.893	0.683	0.607
Immunoglobulin M, g/L	4.32	4.88	4.06	5.60	0.891	0.627	0.455	0.584
Tumor necrosis factor-α, ng/L	61.79	57.61	61.69	75.38	5.632	0.863	0.529	0.574
Interleukin 2, pg/mL	31.76	31.85	28.89	33.62	1.224	0.082	0.632	0.072
Interleukin 4, pg/mL	13.65	14.09	13.14	12.57	1.012	0.741	0.366	0.625
Interleukin 6, pg/mL	74.82	75.09	70.11	76.07	2.351	0.305	0.907	0.239
Interferon -γ, pg/L	184.16	186.82	176.07	177.97	7.172	0.686	0.371	0.958
Myostatin, ng/mL	38.81	39.31	39.17	38.95	2.283	0.999	0.978	0.876
Growth hormone, ng/mL	7.37	8.27	8.67	8.17	0.572	0.445	0.282	0.229
Growth hormone-releasing hormone, pg/mL	31.65	26.13	30.93	27.20	1.792	0.107	0.297	0.623
Insulin-like growth factor-1, ng/mL	230.53	190.06	227.57	190.83	15.412	0.133	0.250	0.905

SEM = standard error of the mean.

Values within a row with no common superscripts differ significantly (*P* < 0.05). All values are based on six lambs per treatment (*n* = 6).

1 ALF, ad libitum feeding; LRF, low restricted feeding; MRF, medium restricted feeding; HRF, high restricted feeding.

### 3.4 Apparent nutrient digestibility

During the restriction period, apparent digestibility of CP (*P* = 0.040) and EE (*P* = 0.023, Table 5) was lower in the MRF and HRF groups than in the ALF group. Apparent NDF digestibility was lower in the MRF and HRF groups than in the ALF and LRF groups (*P* = 0.004) and showed a significant linear decrease with increasing restriction level (*P* = 0.001). During compensatory feeding, apparent CP digestibility was higher in the LRF group than in the ALF and MRF groups (*P* = 0.032). Apparent NDF digestibility was higher in the MRF and HRF groups than in the ALF and LRF groups (*P* = 0.014). ADF digestibility was higher in the MRF and HRF groups than in the ALF group (*P* = 0.045) and increased linearly with increasing restriction level (*P* = 0.032).

**Table 5:** Effects of feed intake-regulated nutritional compensation strategies on the nutrient digestibility (%) in Gangba sheep.

Item	Groups^1^					*P*-value		
	ALF	LRF	MRF	HRF	SEM	Treatment	Linear	Quadratic
**Restricted feeding period**								
Crude protein	65.13^a^	66.58^a^	62.14^b^	62.64^b^	0.192	0.040	0.495	0.379
Ether extract	56.55^a^	51.37^ab^	45.76^c^	48.07^bc^	0.711	0.023	0.076	0.325
Neutral detergent fiber	55.06^a^	55.01^a^	50.33^b^	49.35^b^	1.202	0.004	0.001	0.705
Acid detergent fiber	46.20	44.33	43.08	42.77	1.233	0.219	0.049	0.531
Organic matter	52.92	51.82	51.32	53.12	1.124	0.177	0.056	0.447
**Compensatory feeding period**								
Crude protein	65.45^b^	72.52^a^	67.55^b^	69.68^ab^	0.621	0.032	0.095	0.179
Ether extract	70.70	69.15	64.15	62.01	0.642	0.058	0.465	0.171
Neutral detergent fiber	61.34^b^	61.48^b^	63.51^a^	63.42^a^	0.458	0.014	0.144	0.075
Acid detergent fiber	51.44^b^	53.47^ab^	56.47^a^	55.87^a^	1.841	0.045	0.032	0.147
Organic matter	64.23	66.75	68.45	69.67	1.054	0.104	0.348	0.366

SEM = standard error of the mean.

Values within a row with no common superscripts differ significantly (*P* < 0.05). All values are based on six lambs per treatment (*n* = 6).

1 ALF, ad libitum feeding; LRF, low restricted feeding; MRF, medium restricted feeding; HRF, high restricted feeding.

### 3.5 Gastrointestinal morphology

During the restricted feeding period, ruminal papilla height was lower in the MRF and HRF groups than in the ALF and LRF groups (*P* = 0.006; Table 6) and decreased linearly with increasing restriction intensity (*P* = 0.003). Ruminal papilla surface area was lower in the MRF and HRF groups than in the ALF group (*P* = 0.027) and also decreased linearly with increasing restriction intensity (*P* = 0.015). Similarly, jejunal VH was lower in the MRF and HRF groups than in the ALF and LRF groups (*P* < 0.001) and decreased linearly with increasing restriction intensity (*P* < 0.001).

**Table 6:** Effects of nutritional compensation on the morphology of the rumen and jejunum in Gangba sheep.

Item	Groups^1^					*P*-value		
	ALF	LRF	MRF	HRF	SEM	Treatment	Linear	Quadratic
**Restricted feeding period**								
**Rumen**								
Papillae height, μm	1848.02^a^	1596.76^a^	1099.35^b^	1018.12^b^	166.971	0.006	0.003	0.333
Papillae width, μm	404.82	381.19	415.67	377.23	23.612	0.594	0.975	0.195
Papillae surface area, mm^2^	0.73^a^	0.61^ab^	0.46^b^	0.40^b^	0.083	0.027	0.015	0.269
**Jejunum**								
VH, μm	612.34^a^	568.09^a^	520.45^b^	493.73^b^	15.684	<0.001	<0.001	0.582
CD, μm	257.18	255.00	239.53	227.35	12.971	0.347	0.085	0.704
VH/CD	2.42	2.23	2.20	2.18	0.102	0.294	0.093	0.390
**Compensatory feeding period**								
**Rumen**								
Papillae height, μm	2343.10^a^	2183.16^a^	1580.37^b^	1501.93^b^	173.934	0.005	<0.001	0.817
Papillae width, μm	563.89	496.72	526.21	510.98	41.455	0.695	0.256	0.780
Papillae surface area, mm^2^	1.04^a^	0.87^a^	0.63^b^	0.61^b^	0.082	0.002	<0.001	0.345
**Jejunum**								
VH, μm	668.27^c^	762.25^b^	803.37^a^	883.95^a^	27.341	0.035	0.008	0.905
CD, μm	295.42^b^	268.25^b^	306.20^b^	370.58^a^	11.171	0.015	0.010	0.043
VH/CD	2.34^b^	2.91^a^	2.68^ab^	2.38^b^	0.072	0.012	0.863	0.002

VH = villus height; CD = crypt depth; SEM = standard error of the mean.

Values within a row with no common superscripts differ significantly (*P* < 0.05). All values are based on six lambs per treatment (*n* = 6).

1 ALF, ad libitum feeding; LRF, low restricted feeding; MRF, medium restricted feeding; HRF, high restricted feeding.

During the compensatory feeding period, ruminal papilla height and surface area in the MRF and HRF groups remained significantly lower than those in the ALF and LRF groups and continued to exhibit linear effects (*P* < 0.05). Jejunal CD was highest in the HRF group and showed a quadratic effect (*P* = 0.043). The VH/CD was significantly greater in the LRF group than in the ALF and HRF groups (*P* = 0.012), also showing a quadratic effect (*P* = 0.002).

### 3.6 Rumen fermentation parameters

During the restricted feeding period, ruminal ammonia nitrogen concentration decreased linearly with increasing restriction intensity (*P* < 0.001), with the MRF and HRF groups showing lower levels than the ALF and LRF groups (*P* < 0.001; Table 7). Acetic acid concentration was lower in the HRF group than in the other treatments and showed a linear decline with increasing restriction (*P* < 0.05). Iso-butyric acid and butyric acid concentrations were also reduced in the MRF and HRF groups compared with the ALF group, both exhibiting linear effects (*P* < 0.05). In addition, total VFAs concentration was reduced in the HRF group, with a linear decrease observed as restriction intensified (*P* < 0.05). In addition, during the compensatory feeding period, no significant differences were detected among treatments in ammonia nitrogen, individual VFAs, total VFA concentration, or VFA molar proportions (*P* > 0.05).

**Table 7:** Effects of feed intake-regulated nutritional compensation strategies on the rumen fermentation parameters of Gangba sheep.

Item	Groups^1^					*P*-value		
	ALF	LRF	MRF	HRF	SEM	Treatment	Linear	Quadratic
**Restricted feeding period**								
pH	6.48	6.45	6.44	6.46	0.052	0.946	0.721	0.632
Ammonia nitrogen, mg/L	122.96^a^	73.09^b^	41.87^c^	38.61^c^	5.994	<0.001	<0.001	0.181
Acetic acid, mmol/L	49.40^a^	47.37^a^	48.12^a^	39.14^b^	2.541	0.039	0.016	0.187
Propionic acid, mmol/L	24.31	22.99	21.04	20.57	1.282	0.175	0.033	0.748
Iso-butyric acid, mmol/L	1.60^a^	1.35^ab^	1.24^b^	1.23^b^	0.104	0.049	0.011	0.240
Butyric acid, mmol/L	7.79^a^	7.64^a^	6.89^b^	6.72^b^	0.253	0.013	0.002	0.966
Iso-valeric acid, mmol/L	2.43	2.24	2.11	1.98	0.123	0.076	0.011	0.822
Valeric acid, mmol/L	1.39	1.22	1.37	1.26	0.124	0.668	0.647	0.744
Total volatile fatty acids, mmol/L	86.92^a^	82.81^a^	80.76^a^	70.90^b^	3.201	0.014	0.002	0.380
Acetic acid, %	56.83	57.19	59.49	54.71	1.412	0.157	0.527	0.083
Propionic acid, %	27.82	27.76	26.12	29.31	1.381	0.466	0.652	0.255
Iso-butyric acid, %	1.87	1.63	1.54	1.74	0.131	0.322	0.420	0.099
Butyric acid, %	9.05	9.25	8.52	9.60	0.412	0.330	0.619	0.297
Iso-valeric acid, %	2.83	2.70	2.63	2.82	0.162	0.768	0.897	0.321
Valeric acid, %	1.60	1.47	1.70	1.82	0.161	0.467	0.219	0.431
**Compensatory feeding period**								
pH	6.56	6.52	6.49	6.58	0.061	0.713	0.856	0.284
Ammonia nitrogen, mg/L	62.46	55.50	66.90	49.25	11.372	0.709	0.585	0.644
Acetic acid, mmol/L	86.56	94.07	88.30	93.76	10.293	0.937	0.735	0.921
Propionic acid, mmol/L	28.55	27.06	26.03	26.57	3.341	0.956	0.646	0.764
Iso-butyric acid, mmol/L	3.74	4.05	3.88	3.67	0.534	0.961	0.873	0.636
Butyric acid, mmol/L	2.82	3.24	3.15	3.66	0.681	0.852	0.432	0.954
Iso-valeric acid, mmol/L	5.12	5.42	4.28	4.11	0.681	0.477	0.190	0.731
Valeric acid, mmol/L	1.21	1.00	0.90	0.88	0.122	0.239	0.060	0.459
Total volatile fatty acids, mmol/L	128.00	134.85	126.53	132.65	14.084	0.972	0.930	0.980
Acetic acid, %	68.12	69.50	69.34	70.54	1.643	0.777	0.343	0.959
Propionic acid, %	21.86	20.17	20.89	19.84	1.151	0.620	0.311	0.786
Iso-butyric acid, %	2.92	3.15	3.00	2.90	0.361	0.959	0.898	0.650
Butyric acid, %	2.09	2.23	2.65	2.74	0.391	0.594	0.192	0.957
Iso-valeric acid, %	4.08	4.18	3.37	3.31	0.562	0.585	0.231	0.888
Valeric acid, %	0.94	0.77	0.76	0.67	0.101	0.294	0.074	0.716

SEM = standard error of the mean.

Values within a row with no common superscripts differ significantly (*P* < 0.05). All values are based on six lambs per treatment (*n* = 6).

1 ALF, ad libitum feeding; LRF, low restricted feeding; MRF, medium restricted feeding; HRF, high restricted feeding.

### 3.7 Rumen enzyme activities

During the restricted feeding period, ruminal protease activity was significantly lower in the MRF and HRF groups than in the ALF and LRF groups, showing both linear and quadratic effects (*P* < 0.05; Table 8). Similarly, cellulase activity was reduced in the MRF and HRF groups compared with the ALF and LRF groups and exhibited a linear decrease with increasing restriction intensity (*P* < 0.05). In addition, lysozyme activity was highest in the HRF group and showed a quadratic response to the restriction level (*P* < 0.05). During the compensatory feeding period, none of the measured enzyme activities differed significantly among treatments (*P* > 0.05).

**Table 8:** Effects of feed intake-regulated nutritional compensation strategies on the rumen enzyme activities of Gangba sheep.

Item	Groups^1^					*P*-value		
	ALF	LRF	MRF	HRF	SEM	Treatment	Linear	Quadratic
**Restricted feeding period**								
Protease, U/mL	3.57^a^	1.56^b^	0.96^c^	1.15^c^	0.471	0.003	0.001	0.031
Lipase, U/mL	4.28	4.12	5.43	3.67	0.482	0.092	0.810	0.109
Cellulase^2^, μg/min/mL	10.06^a^	8.74^a^	7.74^b^	7.77^b^	0.591	0.035	0.007	0.264
Lysozyme, μg/mL	20.78^b^	17.99^b^	23.90^ab^	33.55^a^	0.274	0.004	0.064	0.002
α-Amylase^2^, mg/min/L	74.34	74.25	66.55	78.07	3.723	0.201	0.837	0.135
**Compensatory feeding period**								
Protease, U/mL	3.45	2.10	3.23	2.26	0.432	0.264	0.706	0.719
Lipase, U/mL	2.12	2.28	3.45	2.87	0.261	0.275	0.153	0.480
Cellulase, μg/min/mL	11.42	13.20	14.88	10.44	1.312	0.114	0.831	0.028
Lysozyme, μg/mL	20.26	19.45	23.97	18.02	2.753	0.171	0.262	0.361
α-Amylase, mg/min/L	64.51	60.26	70.91	49.25	15.734	0.623	0.623	0.586

SEM = standard error of the mean.

Values within a row with no common superscripts differ significantly (*P* < 0.05). All values are based on six lambs per treatment (*n* = 6).

1 ALF, ad libitum feeding; LRF, low restricted feeding; MRF, medium restricted feeding; HRF, high restricted feeding.

2 Cellulase and α-amylase activities are expressed as the amounts of reducing sugars released during substrate hydrolysis.

### 3.8 Rumen microbiota

In this study, a total of 48 rumen fluid samples were collected during the restricted feeding and compensatory feeding periods for microbial diversity analysis. A total of 3,612,558 optimized sequences were obtained, comprising 5,382,761,451 bases with an average sequence length of 1490 bp. Taxonomic annotation identified 7278 ASVs, belonging to 25 phyla, 141 families, and 720 species.

During the restricted feeding period, a higher degree of feed restriction significantly reduced the α-diversity of the rumen microbiota in Gangba sheep (Fig. 1A–D), as reflected by lower Ace and Chao indices (*P* < 0.05). Principal coordinate analysis (PCoA) analysis further revealed clear separation of microbial communities among groups (*R* = 0.552, *P* = 0.001; Fig. 1E). At the phylum level, restricted feeding significantly altered the relative abundance of Bacteroidota, Pseudomonadota, and Planctomycetota (*P* < 0.05; Fig. S1A–C). At the family level, significant differences were observed in Succinivibrionaceae, Oscillospiraceae, and Aeromonadaceae (*P* < 0.05; Fig. S1D–F). At the species level, a total of 12 taxa were significantly affected, including *Succinivibrio dextrinosolvens*, *Bacteroidetes bacterium 41 to 46*, *Oceanisphaera profunda*, Ruminococcaceae *bacterium AB4001*, *Mariniphaga sediminis*, *Prevotella* sp. *E9-3*, *Marseilla massiliensis*, *Eubacterium ruminantium*, *unclassified c_Gammaproteobacteria*, *Prevotella lacticifex*, *unclassified f_Prevotellaceae*, and *Pedobacter psychrophilus* (*P* < 0.05; Fig. S1G–O).

**Fig. 1:**
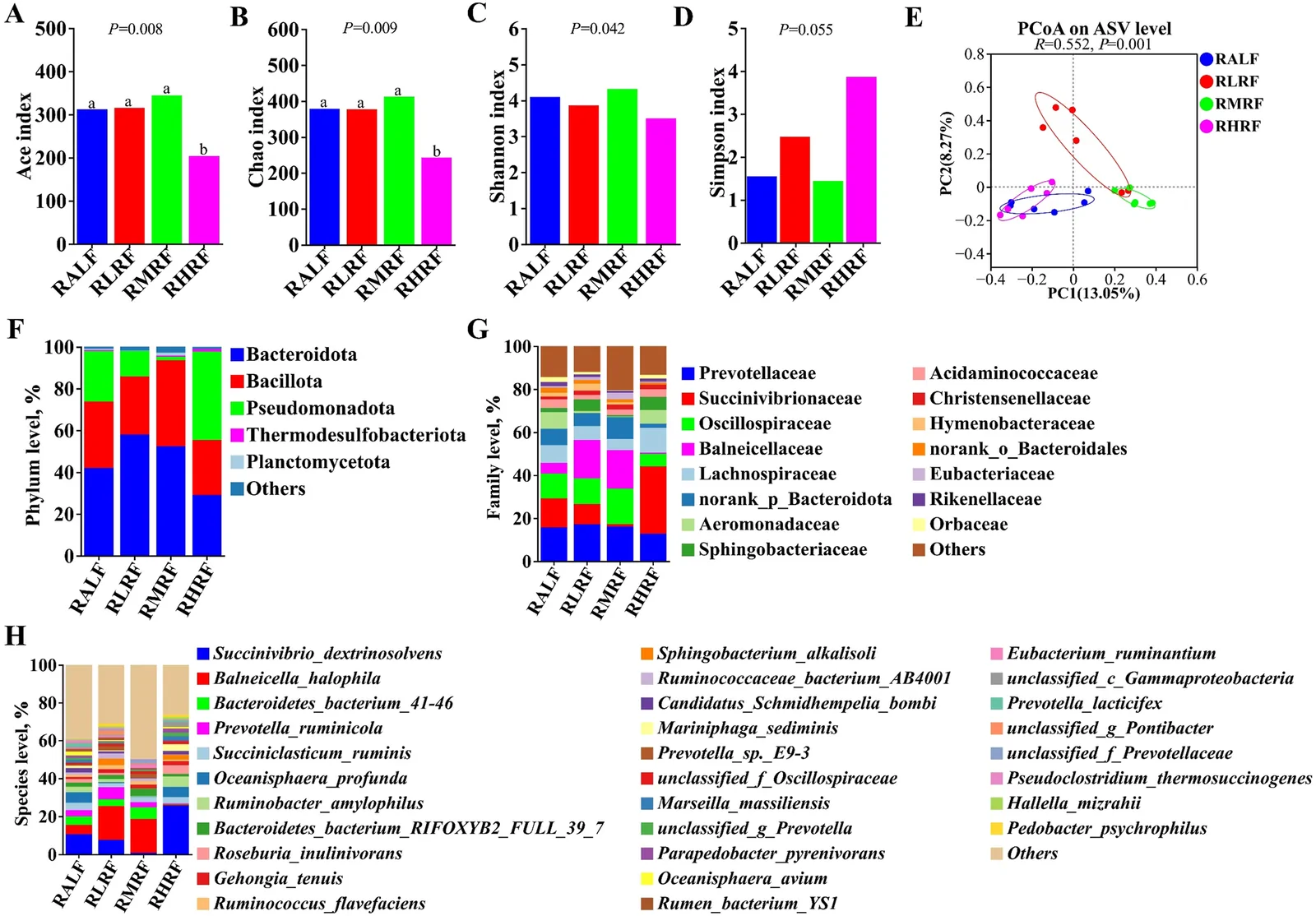
Rumen microbiota diversity during the restricted feeding period. (A) Ace index. (B) Chao1 index. (C) Shannon index. (D) Simpson index. (E) Principal coordinate analysis (PCoA). (F) Microbial composition at the phylum level. (G) Microbial composition at the family level. (H) Microbial composition at the species level. Each treatment included six biological replicates (*n* = 6). Different lowercase letters above bars indicate significant differences among treatments (*P* < 0.05). RALF, ad libitum feeding during the feed restriction period; RLRF, low restriction feeding during the feed restriction period; RMRF, medium restriction feeding during the feed restriction period; and RHRF, high restriction feeding during the feed restriction period. ASV = amplicon sequence variant; PC = principal coordinate.

During the compensatory feeding period, there were no significant differences in Ace, Shannon, or Chao indices (*P* > 0.05), indicating that compensatory feeding did not markedly affect the α-diversity of the rumen microbiota (Fig. 2A–D). However, PCoA analysis showed that microbial community structures still differed significantly among groups (*R* = 0.204, *P* = 0.004; Fig. 2E). At the phylum level, Bacteroidota was the dominant phylum across all four groups (Fig. 2F). At the family level, Prevotellaceae was the dominant phylum across all four groups (Fig. 2G). At the species level, *Oceanisphaera profunda* was dominant in the ALF group, while *Prevotella ruminicola* predominated in the LRF, MRF, and HRF groups (Fig. 2H). Furthermore, LEfSe analysis (LDA > 2; Fig. S2.) identified 16 potential microbial biomarkers. Among them, six taxa were at the species level: *s_Oribacterium parvum* and *s_Ruminococcus albus* in the ALF group; *s_unclassified* and *g_Anaerocolumna* in the MRF group; and *s_Ruminococcoides bili*, *s_Clostridiales bacterium CIEAF_021*, and *s_Bacteroides pyogenes* in the HRF group.

**Fig. 2:**
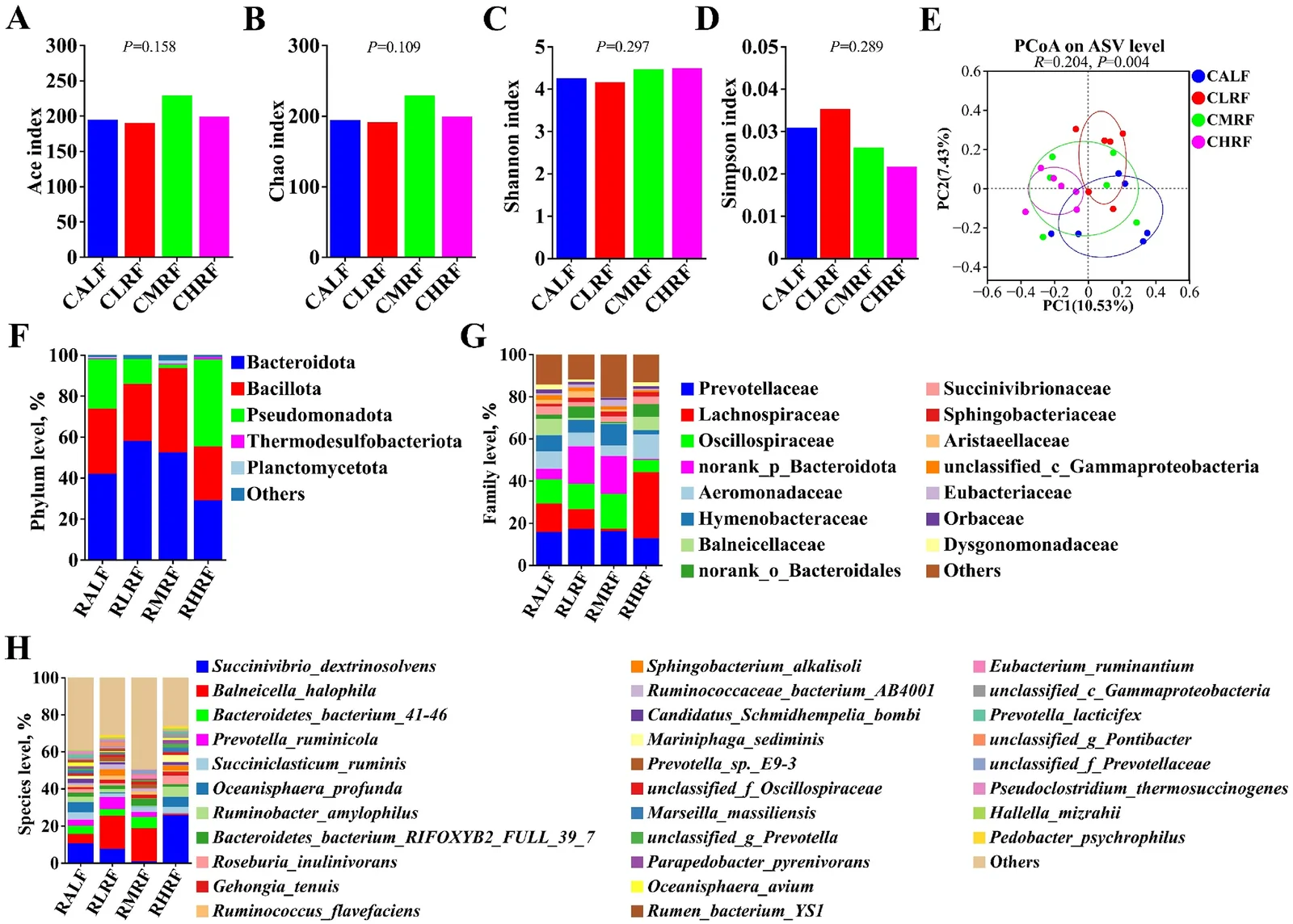
Rumen microbiota diversity during the compensatory feeding period. (A) Ace index. (B) Chao1 index. (C) Shannon index. (D) Simpson index. (E) Principal coordinate analysis (PCoA). (F) Microbial composition at the phylum level. (G) Microbial composition at the family level. (H) Microbial composition at the species level. Each treatment included six biological replicates (*n* = 6). CALF, sheep previously fed ad libitum; CLRF, sheep previously subjected to low feed restriction; CMRF, sheep previously subjected to medium feed restriction; and CHRF, sheep previously subjected to high feed restriction, all during compensatory refeeding. ASV = amplicon sequence variant; PC = principal coordinate.

### 3.9 Spearman correlation analysis

Spearman correlation analysis was performed between differential rumen metabolites, enzyme activities, and the top 30 most abundant microbial taxa (Fig. 3). The results showed that ammonia nitrogen concentration was positively correlated with the relative abundance of *Pseudoclostridium thermosuccinogenes* (*R* = 0.418, *P* = 0.043). Acetic acid concentration was positively associated with the relative abundance of *Succiniclasticum ruminis* (*R* = 0.473, *P* = 0.022) but negatively correlated with the relative abundance of *Sphingobacterium alkalisoli* (*R* = −0.423, *P* = 0.038). Iso-butyric acid concentration was also negatively correlated with the relative abundance of *Sphingobacterium alkalisoli* (*R* = −0.448, *P* = 0.033). Butyric acid concentration showed a positive correlation with the relative abundance of *rumen bacterium YS1* (*R* = 0.461, *P* = 0.024), while total VFA concentration was negatively correlated with the relative abundance of *Sphingobacterium alkalisoli* (*R* = −0.444, *P* = 0.031). Regarding enzyme-related parameters, lysozyme concentration was positively correlated with the relative abundance of *Succinivibrio dextrinosolvens* (*R* = 0.483, *P* = 0.020), *Oceanisphaera profunda* (*R* = 0.483, *P* = 0.047), *Mariniphaga sediminis* (*R* = 0.511, *P* = 0.007), an unclassified *c_Gammaproteobacteria* (*R* = 0.503, *P* = 0.011), and *Prevotella lacticifex* (*R* = 0.532, *P* = 0.008). In contrast, negative correlations were observed with the relative abundance of *Bacteroidetes bacterium 41 to 46* (*R* = −0.541, *P* = 0.006), *Prevotella ruminicola* (*R* = −0.445, *P* = 0.032), Ruminococcaceae *bacterium AB4001* (*R* = −0.433, *P* = 0.030), and *Prevotella* sp. *E9-3* (*R* = −0.497, *P* = 0.011).

**Fig. 3:**
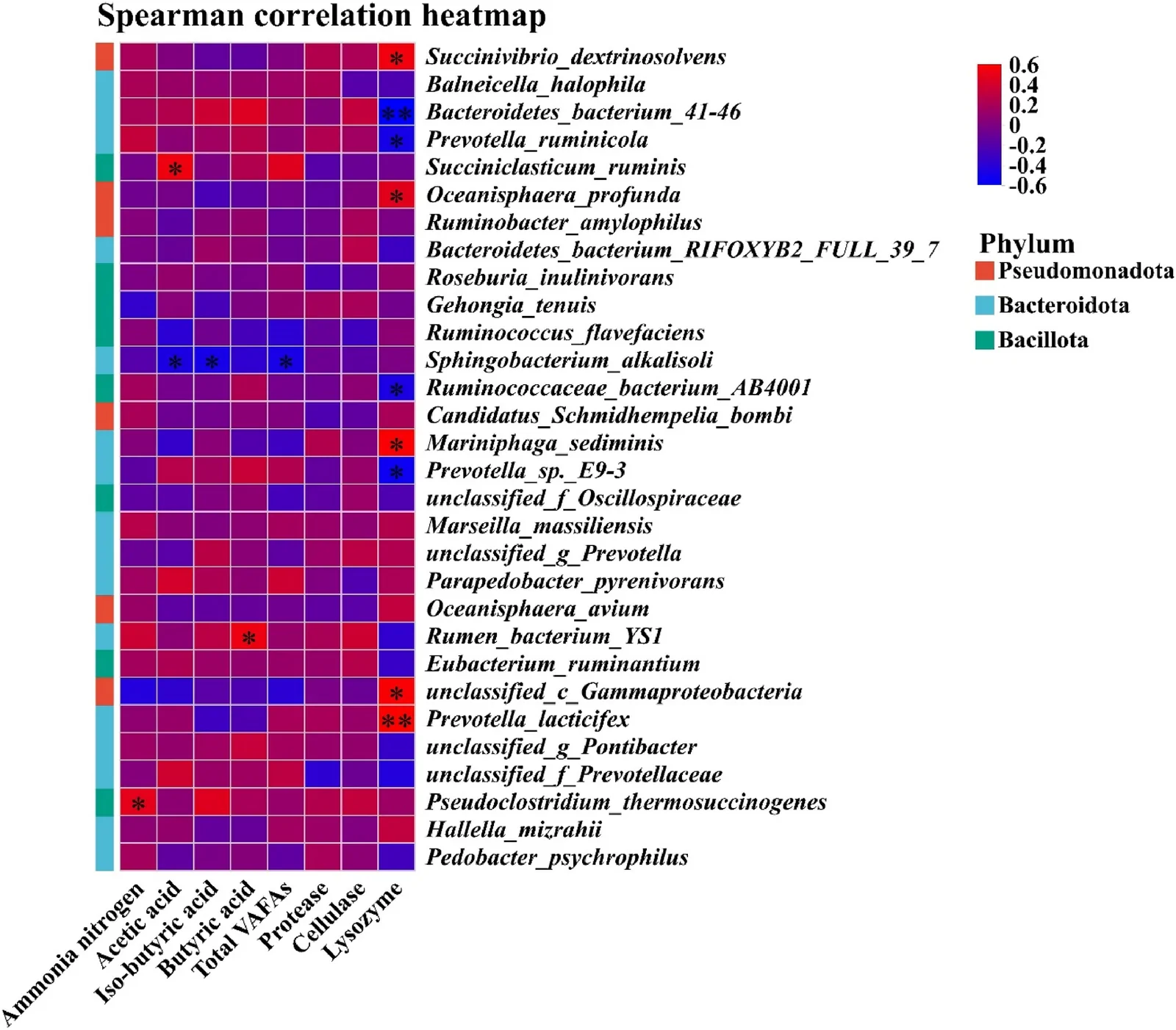
Correlation analysis between rumen microbiota and differential metabolites. Total VFAs is the sum of acetic acid, propionic acid, iso-butyric acid, butyric acid, isovaleric acid, and valeric acid. ∗ *P* < 0.05; ∗∗ *P* < 0.01. VFA = volatile fatty acids.

## 4 Discussion

The present study evaluated how graded feed intake restriction followed by refeeding affected growth performance, nutrient digestion, gastrointestinal morphology, rumen fermentation, and rumen microbiota in Gangba sheep, a high-altitude indigenous breed adapted to chronic forage shortage. Overall, low restriction at 85% of ad libitum intake improved feed efficiency and allowed almost complete recovery of physiological and microbial homeostasis during refeeding, whereas medium (70%) and high (55%) restriction induced sustained impairments in growth, antioxidant and immune status, gastrointestinal structure, and rumen microbial diversity. These findings support the concept proposed in the introduction that the magnitude of prior undernutrition is a critical determinant of compensatory responses and rumen microbial resilience in plateau sheep production systems.

During the restriction period, reductions in BW, ADG, and ADFI were observed with increasing restriction intensity, which is consistent with previous findings in ruminants that nutrient restriction suppresses growth by reducing nutrient supply and altering energy allocation (Ding et al., 2016; Hornick et al., 2000; Neto et al., 2011). During the compensatory period, partial recovery of growth performance occurred, particularly in the LRF group (85% of ALF), which exhibited a better feed conversion ratio than the ALF group over the whole experiment. This pattern agrees with classical compensatory growth theory, where moderate restriction can trigger efficient compensatory growth, whereas excessive undernutrition reduces the capacity to fully compensate despite subsequent re-alimentation (Hornick et al., 2000; Hu et al., 2019; Neto et al., 2011). Similar phenomena have been reported in yaks and other ruminants, where improved nutrition during refeeding enhances growth rate and rumen function mainly when the preceding restriction is not too severe (Ding et al., 2016; Zou et al., 2019). The quadratic response of F: G further suggests the existence of an optimal restriction level that minimizes growth loss while maximizing feed efficiency. In the present study, feeding at 85% of ALF reduced cumulative ADFI while maintaining ADG comparable to ALF, resulting in a 5.48% improvement in F: G, whereas 70% and 55% restriction impaired overall ADG and increased F:G. This indicates that for Gangba sheep, mild intake restriction can be used as a management tool to improve feed efficiency, but restrictions beyond 30% of ad libitum compromise growth potential even after 60 d of refeeding.

The alterations in serum antioxidant and immune indicators during the restriction period provide mechanistic insight into these performance differences. Total antioxidant capacity and SOD activity play critical roles in scavenging free radicals and alleviating oxidative stress (Sies, 2015). In the present study, medium and high restriction decreased T-AOC and SOD levels and increased MDA content, showing a linear deterioration of redox balance with increasing restriction intensity. This suggests that restricted feeding decreases antioxidant enzyme activity and disrupts redox homeostasis, which is in line with reports that nutritional stress in sheep and cattle enhances lipid peroxidation and oxidative damage (Bernabucci et al., 2005; Zhao et al., 2023). Such oxidative stress may result from insufficient antioxidant substrate supply during the restriction period, leading to excessive reactive oxygen species generation (Durand et al., 2022). Importantly, no significant differences in antioxidant indicators were observed among groups during the compensatory phase, indicating that once animals return to ad libitum feeding, body redox balance can be rapidly restored.

Similar dynamic patterns were observed for immune indicators. Immunoglobulins such as IgA and IgM are key components of the adaptive immune system, responsible for recognizing and neutralizing pathogens (Li et al., 2024). During the 30-d restriction phase, serum IgA and IgM levels in the MRF and HRF groups were significantly lower than those in the ALF and LRF groups and decreased linearly with increasing restriction, indicating that the immune system of Gangba sheep was suppressed under severe undernutrition. To maintain basic functions with limited energy and nutrients, the body may downregulate antibody production, thereby increasing susceptibility to infection, which is consistent with previous reports in pigs and small ruminants subjected to malnutrition or mineral imbalance (Wang et al., 2022; Zeng et al., 2021). By the end of the compensatory phase, no differences in IgA and IgM were detected among treatments, suggesting that feed restriction-induced immune suppression is a reversible and transient state and that the immune system can rapidly rebound after nutritional recovery. Similar compensatory immune responses have been reported in other species, such as chickens, where feed restriction exerted complex, sometimes even beneficial, long-term effects on certain immune functions (Orso et al., 2019).

Endocrine changes further support the existence of compensatory physiological mechanisms. The linear increase in serum growth hormone–releasing hormone concentration with restriction intensity in the present study likely reflects activation of the somatotropic axis to mobilize body reserves and support survival during negative energy balance. Growth hormone-releasing hormone (GHRH), secreted by the hypothalamus, stimulates pituitary GH secretion (Huisman et al., 2021); its elevation during restriction can be viewed as an adaptive response aimed at mobilizing body fat and protein to meet energy demands, which is crucial for survival during nutrient scarcity (Ding et al., 2016; Hornick et al., 2000). The absence of differences in GHRH among groups during the compensatory phase confirms that endocrine perturbations induced by restriction can also be normalized after re-alimentation.

The changes in nutrient digestibility and histomorphology provide further mechanistic explanations for the observed differences in growth performance. During the restriction phase, digestibility of CP, EE, and NDF was significantly reduced in the MRF and HRF groups, whereas the LRF group maintained digestibility close to that of ALF. This suggests that moderate restriction may help preserve gastrointestinal function, while more severe restriction impairs digestive efficiency. Although feed restriction is often associated with increased apparent digestibility due to reduced digesta passage rate, the lower digestibility observed in the present study may be related to decreased ruminal microbial activity and limited fermentable substrate supply under severe intake restriction. The marked reduction in ruminal ammonia nitrogen and total VFA concentrations in restricted sheep supports the possibility that impaired microbial growth and fibrolytic activity contributed to reduced nutrient degradation efficiency. These functional changes were closely linked to the morphological adaptations of the rumen and small intestine. It is well established that ruminal papillae height, width, and surface area, as well as greater jejunal VH and lower CD, are positively associated with nutrient absorption efficiency (Hu et al., 2025; Schäff et al., 2018). Because ruminal papillae constitute the primary absorptive surface for VFAs—the main metabolizable energy source in ruminants—reductions in papillae size may directly limit epithelial transport capacity and energy availability for growth, thereby contributing to poorer feed conversion efficiency under severe restriction. In the present study, reductions in ruminal papillae height and surface area and jejunal VH in the MRF and HRF groups likely resulted from decreased VFA, especially butyrate, during restriction. Butyrate is a key trophic factor for rumen epithelial development (Li et al., 2023; Penner et al., 2011; Zhang et al., 2025), and the observed decline in total VFA and butyrate concentration in HRF sheep supports this linkage. During refeeding, CP digestibility increased most in the LRF group, whereas NDF and ADF digestibility were higher in MRF and HRF animals, possibly due to slower passage rate and adaptive changes in fiber-degrading microbiota in previously restricted sheep. During the compensatory feeding phase, the marked increase in fiber digestibility in previously restricted groups may reflect adaptive responses of the rumen ecosystem to prior nutrient limitation. Refeeding after restriction has been reported to stimulate rumen microbial proliferation, enhance fibrolytic activity, and improve rumen epithelial absorptive capacity. These changes may contribute to improved degradation efficiency of structural carbohydrates and partially explain the higher NDF and ADF digestibility observed in the moderate and high restriction groups. Interestingly, although ALF and LRF groups maintained structural advantages in the rumen, MRF and HRF sheep exhibited greater jejunal VH during the compensatory phase, suggesting that ad libitum refeeding may stimulate epithelial remodeling of the small intestine to partially compensate for impaired rumen absorptive capacity. An increased VH/CD is generally associated with a larger effective absorptive surface and lower epithelial turnover cost, which may enhance nutrient assimilation efficiency and partly explain the improved F: G observed under low restriction. Nevertheless, ruminal papillae in MRF and HRF sheep remained shorter with smaller surface area even after 60 d of refeeding, implying incomplete structural recovery of the rumen absorptive surface, which may help explain the reduced growth efficiency despite improved fiber digestibility.

Changes in rumen fermentation parameters and enzyme activities further illustrate how restriction intensity modulated digestive function. During the restricted feeding period, increasing restriction decreased ruminal ammonia nitrogen and total VFA concentrations and reduced iso-butyric and butyric acids, while rumen pH remained within the physiological range. Lower ammonia nitrogen likely reflects both reduced dietary nitrogen supply and impaired proteolysis and deamination, consistent with the decreased protease activity in MRF and HRF groups. The decline in branched-chain VFAs suggests decreased degradation of branched-chain amino acids and potentially reduced microbial protein synthesis, as iso-acids are essential growth factors for many cellulolytic bacteria. Studies in undernourished sheep and calves have similarly reported reduced VFA production and altered fermentation patterns associated with restricted intake or poor-quality diets (Xue et al., 2020). The lower cellulase activity observed in MRF and HRF sheep during restriction corroborates the reduced fiber digestibility and suggests a functional loss of fibrolytic capacity. In contrast, lysozyme activity peaked in HRF sheep, which may reflect increased bacterial turnover or a host attempt to reshape the microbial community under severe nutritional stress. During refeeding, fermentation parameters and enzyme activities converged among treatments, indicating a high degree of functional resilience in rumen fermentation; however, the persistent structural differences in papillae and the incomplete growth recovery suggest that normalization of fermentation alone is not sufficient to guarantee full restoration of digestive function when previous restriction is too severe or prolonged.

Feed restriction and subsequent refeeding also induced marked shifts in rumen microbial communities. During restriction, increasing restriction intensity reduced α-diversity (Ace and Chao indexes) and produced clear separation of microbial communities among treatments on PCoA, indicating that reduced substrate supply acts as a strong ecological filter in the rumen. This is in line with previous studies in sheep and cattle showing that plane of nutrition and undernutrition markedly alter bacterial diversity and the abundance of functional taxa associated with carbohydrate and nitrogen metabolism (Mccabe et al., 2015; Mcgovern et al., 2017; Xue et al., 2020). At the phylum and family levels, restricted feeding modified the relative abundances of Bacteroidota, Pseudomonadota, Planctomycetota, Succinivibrionaceae, Oscillospiraceae, and Aeromonadaceae, while several species—including *Succinivibrio dextrinosolvens*, *Bacteroidetes bacterium 41 to 46*, *Oceanisphaera profunda*, *Prevotella* spp., and Ruminococcaceae *bacterium AB4001*—were particularly sensitive to restriction level. Many of these taxa are involved in starch and fiber degradation, succinate/propionate production, or protein metabolism and are known to respond strongly to dietary energy supply and fermentable carbohydrate availability (Kou et al., 2024; Murillo et al., 2022; Wang et al., 2025).

During the compensatory feeding period, α-diversity indices no longer differed among treatments, suggesting recovery of overall species richness; however, PCoA still showed distinct community structures, and LEfSe identified several treatment-specific biomarkers, indicating that the history of feed restriction left a lasting imprint on community composition. Notably, *Prevotella ruminicola* dominated the restricted groups, whereas *Oceanisphaera profunda* was more abundant in ALF sheep. *Prevotella* spp. are key saccharolytic and proteolytic bacteria capable of degrading starch, hemicellulose, and proteins and producing propionate and succinate as fermentation end-products (Kou et al., 2024; Murillo et al., 2022). Their predominance in restricted groups may reflect a community shift favoring taxa better adapted to efficiently use limited soluble substrates and peptides. In contrast, the reduction of Succinivibrionaceae and other succinate-producing bacteria in some groups is noteworthy, as these taxa participate in succinate–propionate pathways that can improve energy efficiency and hydrogen disposal (Park et al., 2025; Pereira et al., 2022; Shinkai et al., 2024; Stepanchenko et al., 2023). Such shifts may partially contribute to the improved F: G observed in the LRF group, although methane emissions were not measured in this study.

The Spearman correlation analysis provided associative evidence linking specific microbial taxa with fermentation end-products and enzyme activities. Positive correlations between ammonia nitrogen and *Pseudoclostridium thermosuccinogenes*, between acetic acid and *Succiniclasticum ruminis*, and between butyrate and rumen bacterium YS1 may reflect the known or predicted metabolic functions of these taxa in nitrogen turnover and VFA metabolism under the tested nutritional conditions. Meanwhile, negative correlations between *Sphingobacterium alkalisoli* and acetate, propionate, or total VFAs could indicate that certain taxa are relatively enriched under low-fermentation states associated with severe intake restriction. The strong positive correlation between lysozyme activity and several opportunistic or stress-associated taxa (e.g., *Succinivibrio dextrinosolvens*, *Oceanisphaera profunda*, *Mariniphaga sediminis*, and unclassified Gammaproteobacteria), together with negative correlations with *Prevotella ruminicola* and Ruminococcaceae bacterium AB4001, may reflect complex host–microbiota interactions during nutritional stress rather than direct antimicrobial targeting. However, these correlations should be interpreted with caution because they may also arise from shared environmental drivers, compositional constraints inherent to relative abundance data, or multiple-testing effects. Similar microbe–host–metabolite associations have been reported in undernourished sheep and yaks, where altered rumen microbiota–epithelium cross-talk is linked to disrupted gastrointestinal homeostasis (Hu et al., 2019; Wu et al., 2023, 2025; Xue et al., 2020). In addition to dominant rumen bacteria, several non-canonical taxa were differentially abundant across restriction levels. Although their metabolic roles remain unclear, their enrichment under restricted feeding likely reflects ecological filtering driven by reduced substrate availability and host physiological shifts. These taxa may represent opportunistic or stress-tolerant populations with greater metabolic flexibility. This finding highlights the sensitivity of the high-altitude rumen microbiome to nutritional management and warrants further functional investigation.

Taken together, these findings support a mechanistic framework in which restricted feeding alters rumen microbial communities and fermentation patterns, leading to impaired epithelial structure, reduced nutrient absorption, compromised antioxidant capacity, suppressed immune function, and ultimately decreased growth performance. Importantly, feeding at 85% of ad libitum intake appeared to balance physiological homeostasis while improving feed efficiency and achieving favorable compensatory growth upon refeeding. In contrast, 70% and 55% restriction disrupted redox balance and immunity, impaired papilla and villus development, reduced microbial richness, and reshaped rumen community structure in ways that were only partially reversible, leading to poorer overall growth performance. From a practical perspective, implementing mild intake restriction during periods of forage shortage, followed by adequate refeeding, could improve feed efficiency and reduce feed costs in high-altitude production systems without compromising animal health. However, more severe or prolonged restriction should be avoided, as it may cause long-lasting damage to gastrointestinal function and limit compensatory potential. Future studies combining metagenomics and metabolomics with measurements of methane emissions will be valuable to clarify how specific microbial and metabolic pathways mediate the trade-off between feed efficiency, environmental impact, and animal robustness under intake-regulated nutritional strategies in high-altitude indigenous sheep.

## 5 Conclusions

Feed restriction markedly altered growth performance, systemic physiology, and rumen microbial ecology in Gangba sheep. Severe restriction impaired nutrient utilization, antioxidant and immune capacity, as well as gastrointestinal development, resulting in persistent growth suppression even after refeeding. By contrast, mild restriction at 85% of ad libitum intake maintained microbial homeostasis and mucosal integrity while enhancing feed efficiency during the compensatory phase. Together, these results indicate that properly regulated mild feed restriction followed by re-alimentation is a practical nutritional strategy to improve feed efficiency and support sustainable Gangba sheep production under high-altitude, forage-limited conditions.

## Credit Author Statement

**Yining Xie:** Writing – original draft, Validation, Project administration. **Xiaokang Jing:** Methodology, Data curation. **Zhaohan Zhan:** Visualization, Data curation. **Liang Chen:** Writing – review & editing, Supervision, Funding acquisition. **Hongfu Zhang:** Resources, Funding acquisition, Conceptualization.

## Declaration of competing interest

We declare that we have no financial and personal relationships with other people or organizations that can inappropriately influence our work, and there is no professional or other personal interest of any nature or kind in any product, service and/or company that could be construed as influencing the content of this paper.
