# Supplementary material for: Effects of feed intake-regulated nutritional compensation strategies on growth performance, blood indices, and rumen microbiota of Gangba sheep
Source: Anim Nutr. 2026 Aug 4;27:351–64. doi: 10.1016/j.aninu.2026.04.005 (PMC13656949; doi:10.1016/j.aninu.2026.04.005)
Supplement: Supplementary file 1 [file mmc1.docx]

**Fig. S1.** Differential rumen microbial taxa at the phylum, family, and species levels during the feed restriction period. (A–C) Differential taxa at the phylum level. (D–F) Differential taxa at the family level. (G–R) Differential taxa at the species level. RALF, ad libitum feeding during the feed restriction period; RLRF, low restriction feeding during the feed restriction period; RMRF, medium restriction feeding during the feed restriction period; and RHRF, high restriction feeding during the feed restriction period. Different lowercase letters above bars indicate significant differences among treatments (*P* < 0.05). Values are based on six biological replicates per treatment (*n* = 6).

**Fig.** **S2**. Linear discriminant analysis effect size (LEfSe) analysis of rumen microbiota during the compensatory refeeding period. (A) Cladogram showing taxa differentially enriched among treatments. (B) Linear discriminant analysis (LDA) scores of differential taxa. Colors indicate the treatment in which each taxon was enriched. CALF, sheep previously fed ad libitum; CLRF, sheep previously subjected to low feed restriction; CMRF, sheep previously subjected to medium feed restriction; and CHRF, sheep previously subjected to high feed restriction, all during compensatory refeeding. Only taxa with an LDA score > 2.0 and *P* < 0.05 are shown.
